# OpsSatNet: A Lightweight Sensor-Aware Attention Network for Satellite Telemetry Anomaly Detection

**DOI:** 10.3390/s26196126

**Published:** 2026-09-27

**Authors:** Yi Peng, Shuze Jia, Xiaohu Feng, Zeqiu Chen, Xiaodan Zhang

**Affiliations:** 1 National Satellite Meteorological Center (National Center for Space Weather)/Innovation Center for FengYun Meteorological Satellite (FYSIC)/Key Laboratory of Radiometric Calibration and Validation for Environmental Satellites, China Meteorological Administration, Beijing 100081, China; 2 College of Computer Science, Beijing University of Technology, Beijing 100124, China

**Keywords:** satellite telemetry, anomaly detection, intelligent sensors, condition monitoring, sensor-aware attention network

## Abstract

Satellite housekeeping systems combine heterogeneous sensor channels that require continuous fault monitoring. Existing methods rely mainly on hand-crafted statistics or generic raw-signal models, which can obscure localized morphology and do not explicitly adapt shared features to sensor type. They also rarely assess accuracy together with resource cost and temporal evidence, limiting deployment relevance. To address these limitations, we present OpsSatNet, a lightweight sensor-aware attention network for segment-level anomaly detection from raw, resampled telemetry. Specifically, a dilated residual convolutional backbone captures multi-scale patterns, the feature-wise linear modulation adapts shared features by sensor type, and additive attention pooling aggregates temporal features with learned weights. On OPSSAT-AD, the base model achieves an F1-score of 0.960±0.009 over five runs, compared with 0.946 for the strongest published baseline and 0.949±0.004 for the strongest retrained raw-signal baseline. It contains 258K parameters, while a 16K parameter variant retains an F1-score of 0.952±0.010 at 8 MFLOPs and 1.157 ms single-thread CPU latency per segment. Ablations show positive mean F1 differences for sensor conditioning and attention pooling, although neither individual effect is statistically significant. These results support lightweight, sensor-aware monitoring, while cross-mission and flight-hardware validation remain necessary.

## 1. Introduction

Spacecraft housekeeping telemetry is derived from measurements acquired by a distributed sensing system that monitors currents, temperatures, illumination, attitude, and other physical quantities. Timely interpretation of these signals is essential for detecting sensor degradation and subsystem faults before they affect spacecraft availability or safety. Conventional monitoring relies on fixed out-of-limits rules and expert review, but fixed limits are poorly suited to shape-based or context-dependent anomalies, while manual inspection becomes difficult as the number of spacecraft and telemetry channels grows. Short contact windows and constrained downlink further motivate automated processing close to the data source [1,2]. Satellite telemetry anomaly detection is therefore both a condition-monitoring problem and an edge intelligence problem for heterogeneous sensor data.

Machine learning has addressed spacecraft telemetry through forecasting, reconstruction, and direct classification. Representative systems include long short-term memory (LSTM) forecasting with dynamic thresholds [3], stochastic recurrent models [4], reconstruction-based detectors [5,6], and attention-based architectures [7,8]. Progress has nevertheless been limited by scarce public telemetry with expert labels and by disputed evaluation practices on older corpora [9]. The OPSSAT-AD benchmark [1,10] provides segmented, expert-annotated telemetry from ESA’s OPS-SAT CubeSat together with a fixed split and a broad baseline suite, while the ESA Anomaly Benchmark extends public evaluation to several missions [11]. Equipment fault diagnosis has developed from expert-defined indicators and feature-based classifiers toward learned representations, self-supervision, and simulation-assisted training. Recent examples include cross-sensor self-contrastive learning (CSSCL) for label-scarce locomotive-motor fault detection [12], and digital twin-assisted graph contrastive domain adaptation (GCDA) for small-sample bearing diagnosis [13]. These studies provide context for label scarcity and sensor heterogeneity, whereas the present work focuses on supervised anomaly detection in satellite telemetry.

Three gaps remain important from a sensor-systems perspective. First, the strongest published OPSSAT-AD classifiers use hand-crafted statistics rather than the measured signal itself [1]. These features are effective, but they may discard localized morphology and require adaptation when new sensor types are introduced. Second, magnetometer and photodiode channels have distinct nominal shapes and operating ranges, yet a deployable monitor should share most of its parameters across sensor types rather than maintain a separate model for each one. Third, compactness and interpretability are rarely evaluated together with detection accuracy. Resource use matters for edge deployment, and operators need to identify the temporal evidence behind an alarm rather than receive only a binary label.

We hypothesize that a compact raw-signal architecture can address these requirements jointly if it combines multi-scale temporal feature extraction, explicit sensor type conditioning, and attention-based aggregation. We therefore propose OpsSatNet, a lightweight sensor-aware attention network for supervised segment-level telemetry anomaly detection. A dilated residual backbone [14,15] captures localized events and longer temporal context; feature-wise linear modulation (FiLM) [16] makes the network sensor-aware by adapting shared intermediate features to the physical sensor type; and additive attention pooling [17] emphasizes informative timesteps while exposing the weights used for aggregation. Here, lightweight refers to parameter and arithmetic cost, particularly for the tiny variant, and does not imply validated flight deployment. The main contributions of this paper are as follows:

- OpsSatNet combines dilated residual convolutions with FiLM conditioning so that one compact model can learn from magnetometer and photodiode telemetry without hand-crafted input features or separate type-specific networks.
- We report benchmark metrics, parameter counts, floating-point operations, desktop CPU/GPU latency, ablations, per-channel errors, cross-type transfer, and qualitative attention profiles.
- We compare against published feature-based methods, four reproduced feature-based classifiers, and six raw-signal neural baselines evaluated on the same test split over five training seeds, with training-protocol differences. OpsSatNet obtains the highest F1-score among the evaluated methods.

## 2. Related Work

### 2.1. Anomaly Detection in Spacecraft Telemetry

Anomaly detection is a long-standing research area with mature general-purpose taxonomies [18,19]. In the spacecraft domain, the seminal work of Hundman et al. [3] introduced LSTM forecasting with nonparametric dynamic thresholding for NASA’s SMAP and MSL missions and released the accompanying telemetry corpora, which became de facto standards for evaluation. Follow-up work on multivariate telemetry includes OmniAnomaly, which combines stochastic recurrent networks with planar normalizing flows [4], and MSCRED, which detects and diagnoses anomalies through multi-scale signature matrices and convolutional recurrent encoder–decoders [20]. However, later analyses identified substantial flaws in the labeling and the commonly used point-adjustment evaluation of these corpora, showing that trivial detectors can attain state-of-the-art scores under such protocols [9], which motivated the community to seek better-curated benchmarks.

For satellite telemetry specifically, two recent ESA-affiliated benchmarks address this need. The OPSSAT-AD dataset [1,10,21] provides 2123 expert-labeled segments of real telemetry from the OPS-SAT CubeSat with a fixed stratified train/test split. Its reference study evaluates 30 supervised and unsupervised detectors—drawn largely from the PyOD library [22] and including isolation forests [23], random forests [24], XGBoost-based ensembles [25], and generative adversarial detectors [26]—on eighteen hand-crafted statistical features per segment. The best reported result is a fully connected neural network with F1 =0.946. The larger ESA Anomaly Benchmark [11] extends public evaluation to multi-year telemetry from three full-size ESA missions. Subsequent OPSSAT-AD work has examined Transformer and recurrent-attention architectures [27], while recent work on ESA-ADB has used multi-objective architecture optimization to compress detectors for spacecraft edge processors [28]. These studies advance accuracy or efficiency, but do not jointly evaluate raw-signal learning, sensor type adaptation, computational cost, and descriptive temporal weight profiles on OPSSAT-AD.

### 2.2. Machine Learning for Sensor Time-Series Anomaly Detection

Beyond the spacecraft domain, deep learning approaches to time-series anomaly detection include reconstruction, forecasting, and representation learning. LSTM encoder–decoders detect anomalies through reconstruction error [6]; DeepAnT uses convolutional forecasting [29]; USAD trains adversarially coupled autoencoders for fast training on multivariate series [5]; and Transformer-based detectors such as the Anomaly Transformer, which exploits association discrepancy [7], and TranAD, which combines self-conditioning with adversarial training [8], represent the current attention-based state of the art.

Because OPSSAT-AD is formulated as segment-level binary classification, the time-series classificationliterature is equally relevant. Strong convolutional baselines—fully convolutional networks and residual networks—were established by Wang et al. [30], and the comprehensive review of Ismail Fawaz et al. [31] confirmed convolutional architectures as the dominant family. InceptionTime further improved accuracy through multi-scale inception modules [32], while ROCKET showed that random convolutional kernels with linear classifiers achieve competitive accuracy at a fraction of the training cost [33]. Dilated temporal convolutions [14,34] expand context without recurrence; our same-padded encoder is noncausal and processes a complete segment. FiLM [16] provides conditioning, whereas additive attention [17] supplies learned pooling. Transformer self-attention [35] is a different sequence-modeling mechanism and provides one comparison architecture.

### 2.3. Edge AI and Interpretable Sensor Monitoring

Processing sensor data at the edge reduces communication demand and response latency, but requires explicit control of memory, arithmetic cost, and power consumption. Efficient architecture principles such as depthwise separable convolutions, width scaling, and direct accuracy–latency evaluation have shaped the broader edge-AI literature [36]. Space-qualified and CubeSat-class processors impose especially tight constraints, and onboard AI studies consistently identify model footprint, deterministic latency, and hardware validation as central deployment criteria [2]. Consequently, reporting detection accuracy alone is insufficient for assessing whether a telemetry monitor is practically deployable.

Interpretability is important for decision support, but an accessible intermediate weight is not automatically a faithful explanation. Grad-CAM [37] and LIME [38] provide post hoc attributions with additional computation. Attention weights are already available during aggregation, yet Jain and Wallace [39] showed that different weight distributions can yield similar predictions. Wiegreffe and Pinter [40] emphasize that explanatory claims depend on their definition and require controlled tests. Accordingly, we visualize attention as a description of temporal feature weighting.

In summary, prior work provides curated telemetry benchmarks, strong convolutional time-series classifiers, conditioning and attention mechanisms, and established edge-AI design principles. However, the OPSSAT-AD literature has not jointly evaluated raw-signal detection, sensor heterogeneity, computational cost, and descriptive attention profiles within one model. This gap motivates the design presented next.

## 3. Methods

OpsSatNet is the sensor-aware attention network proposed in this work: sensor-aware denotes type-conditioned feature modulation in the shared encoder, whereas attention denotes the learned temporal weighting used for feature aggregation. Figure 1 summarizes its three principal processing stages: input adaptation, sensor-aware temporal feature extraction, and attention-based anomaly classification.

### 3.1. Problem Formulation

Let a telemetry segment be a univariate, irregularly sampled time series

(1) $$S={\left\{\left(t_{i},v_{i}\right)\right\}}_{i=1}^{n},\;t_{1}<t_{2}<\cdots <t_{n},$$

where $v_{i}\in R$ is the telemetry value at timestamp $t_{i}$ and the length *n* varies from segment to segment. Each segment originates from one telemetry channel c∈C, and each channel has a physical sensor type g(c)∈{0,1} (in OPSSAT-AD: magnetometer or photodiode). Each segment carries a binary label y∈{0,1}, where y=1 denotes an anomalous segment as annotated by spacecraft operation engineers. The task is to learn a classifier

(2) $$f_{\theta }:\left(S,g\left(c\right)\right)\mapsto \hat{p}\in \left[0,1\right]$$

that estimates the probability that segment *S* is anomalous, using only segments from the training split for parameter estimation.

### 3.2. Preprocessing

OPSSAT-AD segments have variable lengths and sampling patterns, whereas the convolutional backbone expects a fixed number of input positions. We resample each segment to *T* positions by linear interpolation over normalized sample index, as implemented in the experiment code. Specifically, $x_{\mathrm{old},i}=\left(i-1\right)/\left(n-1\right)$ and $x_{\mathrm{new},j}=\left(j-1\right)/\left(T-1\right)$. Original timestamp spacings are not used in this interpolation:

(3) $$\tilde{v}=\mathrm{Interp}\left(x_{\mathrm{new}};\;x_{\mathrm{old}},v\right)\in R^{T}.$$

This aligns sequence lengths but does not preserve physical duration or irregular time gaps; interpolation can also smooth short events. Each resampled segment is standardized instance-wise,

(4) $$x=\frac{\tilde{v}-\mu \left(\tilde{v}\right)}{\sigma (\tilde{v})+\epsilon },$$

removing per-segment offset and scale so that the network focuses on signal morphology. We use T=256 throughout.

### 3.3. Dilated Residual Convolutional Backbone

The backbone is a stack of *D* residual blocks operating on the normalized input $x\in R^{1\times T}$. Block ℓ∈{1,…,D} applies two same-padded one-dimensional convolutions with kernel size k=7 and dilation $d_{\ell }=2^{\ell -1}$, each followed by batch normalization [41]; a 1 × 1 convolutional shortcut aligns channel widths when they differ:

(5) $$\begin{array}{cc} u & =\mathrm{BN}\left({\mathrm{Conv}}_{k,d_{\ell }}\left(h^{\left(\ell -1\right)}\right)\right), \end{array}$$

(6) $$\begin{array}{cc} h^{\prime } & =\mathrm{BN}\left({\mathrm{Conv}}_{k,d_{\ell }}\left(\mathrm{ReLU}\left(u\right)\right)\right), \end{array}$$

(7) $$\begin{array}{cc} h^{\left(\ell \right)} & =\mathrm{ReLU}\left({\mathrm{FiLM}}_{\ell }\left(h^{\prime }\right)+{\mathrm{Conv}}_{1\times 1}\left(h^{\left(\ell -1\right)}\right)\right), \end{array}$$

where $h^{\left(0\right)}=x$ and channel widths double per block (16,32,64,128 in the base variant, D=4; 8,16,32 in the tiny variant, D=3). Doubling the dilation at each block expands the receptive field exponentially with depth [14,34]: with k=7 and D=4, the theoretical receptive field spans up to 181 input positions—most of the 256-point segment—with a shallow, compact backbone. Residual connections [15] stabilize optimization at this depth.

### 3.4. Sensor Type Conditioning with FiLM

The nine OPSSAT-AD telemetry channels belong to two sensor types. Their different observed profiles motivate type-conditioned feature extraction. We provide a mechanism for such conditioning through FiLM [16], while retaining a shared encoder. Sensor type is known metadata rather than an inferred fault label; no individual channel identifier enters the model. A learned embedding $E_{\ell }:\left\{0,1\right\}\to R^{2C_{\ell }}$ maps the type indicator g(c) to scale and shift vectors:

(8) $$\left(\gamma_{\ell },\beta_{\ell }\right)=E_{\ell }\left(g(c)\right),\;{\mathrm{FiLM}}_{\ell }\left(h^{\prime }\right)=\left(1+\gamma_{\ell }\right)⊙h^{\prime }+\beta_{\ell },$$

where ⊙ denotes channel-wise multiplication broadcast over time. The embedding is zero-initialized, so conditioning starts as the identity and the network learns type-specific modulation only insofar as it reduces the loss. For two types, its parameter cost is $4\sum_{\ell }C_{\ell }$: 960 parameters (0.37% of the base model) or 224 parameters (1.37% of the tiny model). Section 4.6 evaluates the contribution of conditioning, and Section 4.9 examines transfer to an unseen type.

### 3.5. Attention Pooling and Temporal Weight Profiles

The backbone output $H=\left[h_{1},\ldots ,h_{T}\right]\in R^{C_{D}\times T}$ must be reduced to a fixed-size vector. Global average pooling weights all timesteps equally, although anomalies are typically localized events occupying a small fraction of the segment. We instead use additive attention pooling [17]: a two-layer 1 × 1 convolutional scorer assigns each timestep a scalar energy, normalized by a softmax,

(9) $$e_{t}=w^{⊤}\tanh\left(Wh_{t}\right),\;\alpha_{t}=\frac{\exp(e_{t})}{\sum_{s=1}^{T}\exp\left(e_{s}\right)},\;z=\sum_{t=1}^{T}\alpha_{t}\;h_{t},$$

Here, $W\in R^{\left(C_{D}/2\right)\times C_{D}}$ and $w\in R^{C_{D}/2}$; biases are omitted from the notation. The pooled vector *z* feeds the classifier. The normalized weights α indicate how encoded features are aggregated, not how much an original sample causally contributes to the output. Each $h_{t}$ encodes information from up to 181 input positions in the base model; thus, a weight at position *t* is not a pointwise attribution to $x_{t}$. The weights are available from the same forward pass and are visualized in Section 4.10.

### 3.6. Classification Head and Training Objective

A two-layer MLP with dropout maps *z* to a single logit, and $\hat{p}=\sigma \left(\mathrm{logit}\right)$. Because roughly 20% of training segments are anomalous, we optimize class-weighted binary cross-entropy,

(10) $$L\left(\theta \right)=-\frac{1}{N}\sum_{j=1}^{N}\left[\;w_{+}\;y_{j}\log{\hat{p}}_{j}+\left(1-y_{j}\right)\log\left(1-{\hat{p}}_{j}\right)\right],\;w_{+}=\frac{N_{\mathrm{neg}}}{N_{\mathrm{pos}}},$$

where $w_{+}\approx 4$ counteracts the class imbalance.

## 4. Experiments

### 4.1. Dataset and Benchmark

OPSSAT-AD [1,21] contains real telemetry from European Space Agency (ESA)’s OPS-SAT, a 3U CubeSat operated as an in-orbit experimentation laboratory from 2019 to 2024. The dataset comprises 303,493 raw telemetry samples organized into 2123 variable-length segments drawn from nine channels of two sensor types: three magnetometer telemetry channels (I_B_FB_MM_0/1/2) and six photodiode elevation channels (I_PD1_THETA–I_PD6_THETA). Segments were manually delimited and labeled anomalous or nominal by three ESA spacecraft operation engineers, with curation by two machine learning experts. The official stratified split fixes 1594 training segments (1273 nominal, 321 anomalous) and 529 test segments (416 nominal, 113 anomalous), an overall anomaly rate of 20.4%. We re-derived all of these statistics independently from the raw files and confirmed exact agreement with the published values; we use the official split unmodified. Table 1 summarizes the per-channel composition, and Figure 2 shows representative nominal and anomalous segments where available for each channel. Magnetometer channels (top row) exhibit smooth quasi-sinusoidal nominal profiles, with anomalies appearing as spikes, dropouts, and flat plateaus; photodiode channels (rows two and three) show bounded illumination profiles, with anomalies manifesting as distorted shapes, bursts, and timing shifts.

Segment lengths range from 8 to 1040 samples, with a median of 70 (interquartile range 40–201), and sampling periods differ across channels. These statistics motivate fixed-length resampling, with the limitations stated in Section 3.2. Anomaly incidence also varies by channel: it is 0% for I_PD1_THETA, 35.6% for I_B_FB_MM_2, and 78.6% for the sparsely represented I_PD4_THETA. Per-channel behavior therefore requires separate analysis (Section 4.8).

The anomaly label is binary at segment level. Observed morphologies include unusual shapes, isolated or repeated peaks, noisy or distorted profiles, zero-valued intervals, irregular periodicity, and gaps. These are descriptive examples rather than mutually exclusive annotated fault classes; no per-fault-type counts or confirmed physical root causes are available in the labels used here.

According to the provided segment-level train flag, all samples of each segment in its assigned partition were retained rather than splitting individual samples or windows. The 1594/529 split is approximately 75/25; no additional random train/test split is drawn. The main models use all 1594 training segments. Only the recurrent and cross-type protocols create a training-derived subset of 1275 fitting and 319 validation segments; the 529 test segments remain unchanged. The official split is not a chronological holdout or a held-out mission/type, so its results characterize within-benchmark generalization and do not establish temporal independence between all source recordings.

### 4.2. Experimental Setup

Models are implemented in PyTorch 2.5.1 with CUDA 12.1 and trained on one (NVIDIA Corporation, Santa Clara, CA, USA) GeForce RTX 4090 (24 GB). The experiment host has an Intel Core i9-10900X CPU (Intel Corporation, Santa Clara, CA, USA) (10 physical cores, 20 logical processors, nominal 3.70 GHz), 62.5 GiB OS-visible RAM, and Ubuntu 20.04.6 LTS (Canonical Group Limited, London, UK). Unless noted otherwise, every configuration uses the hyperparameters of Table 2 and is trained five times with seeds {42,1,7,123,2024}; we report mean ± standard deviation across runs. The primary protocol uses all 1594 official training segments for a predeclared budget of 60 epochs and evaluates the 529-segment test partition once at the final epoch with a fixed threshold of 0.5. Thus, neither checkpoint nor threshold selection uses test performance. Recurrent models receive additional training-only tuning: deterministic channel–label stratification (split seed 2025) produces 1275 fitting and 319 validation segments, the learning rate is chosen from $\left\{10^{-3},3\times 10^{-4}\right\}$ by mean validation F1, and the validation-best checkpoint is tested without refitting. These different training protocols should be considered when comparing architectures.

### 4.3. Evaluation Metrics

We adopt the seven metrics prescribed by the OPSSAT-AD benchmark [1]: accuracy, precision, recall, F1-score, Matthews correlation coefficient (MCC), area under the ROC curve (AUC-ROC), and area under the precision–recall curve (AUC-PR). With TP, FP, TN, FN denoting the confusion-matrix entries at a decision threshold of 0.5,    

(11) $$\mathrm{Precision}=\frac{TP}{TP+FP}\;,\;\mathrm{Recall}=\frac{TP}{TP+FN}\;,\;F1=\frac{2\;TP}{2\;TP+FP+FN}\;,$$

(12) $$\mathrm{MCC}=\frac{TP\cdot TN-FP\cdot FN}{\sqrt{\left(TP+FP)(TP+FN)(TN+FP)(TN+FN\right)}}.$$

MCC incorporates all four confusion-matrix entries, while the two AUC metrics characterize threshold-free ranking quality. F1 on the anomalous class is the benchmark’s primary ranking criterion, which we follow.

For each comparison, we pair F1 values by training seed and apply a two-sided paired *t* test with four degrees of freedom, reporting a 95% confidence interval for the mean difference. The paired *t* test assumes approximately normal paired differences, an assumption that is difficult to assess with five runs. These tests measure run-to-run variation conditional on one fixed dataset split, not uncertainty across independent missions or test sets. We additionally report Holm-adjusted *p* values across the six retrained raw-signal baselines. The archived per-run F1 values are rounded to three decimals, so near-threshold *p* values should not be interpreted as precise evidence. A two-sided exact sign-flip sensitivity check enumerates all $2^{5}$ signs of the paired differences. With only five pairs, its minimum attainable *p* value is 0.0625. The comparisons are exploratory, and a failure to reject the null is not evidence of equivalence.

### 4.4. Baselines

We compare against three groups of baselines.

**(1) Published feature-based baselines.** The original benchmark evaluated 30 detectors on eighteen hand-crafted features [1]; we report results for the strongest representatives: the FCNN (best overall, F1 =0.946), XGBOD, RF + ICCS, and the best unsupervised method, MO-GAAL [26].

**(2) Reproduced feature-based baselines.** As a sanity check of our split and metric pipeline, we retrained AdaBoost, random forest [24], XGBoost [25], and an MLP on the same eighteen features, following the reference implementation. Our random forest and XGBoost obtain F1 =0.921 and 0.908, respectively; these models differ from the RF + ICCS and XGBOD configurations reported in the original benchmark. The untuned feature-based MLP obtains F1 =0.749.

**(3) Raw-signal deep baselines.** To isolate the contribution of our architecture rather than the raw-signal input format, we retrained six standard deep architectures using the same resampled inputs and loss function, with the same five random seeds: an MLP on the flattened signal; a plain three-layer CNN with max pooling (no residuals, dilations, FiLM, or attention); LSTM, BiLSTM, and GRU recurrent classifiers [42]; and a patch-based Transformer encoder with a classification token [35]. The non-recurrent baselines use the same fixed-budget protocol as OpsSatNet; the recurrent baselines use the more favorable training-only validation procedure described above.

### 4.5. Overall Detection Performance

Table 3 reports the main comparison. OpsSatNet-base attains the highest mean F1 (0.960±0.009) and MCC (0.950±0.011) among the evaluated models. Recurrent classifiers remain variable despite validation-based tuning, while the plain CNN is the strongest raw-signal baseline at F1 =0.949±0.004. The paired mean F1 advantage over CNN is 0.0118, with t(4)=2.79 and nominal p=0.0495 (95% CI: 0.00004–0.02356). However, Holm correction across six raw-signal baselines yields p=0.0688, and an exact two-sided sign-flip test gives p=0.0625. Thus, the CNN comparison does not provide robust evidence at the 0.05 level. Table 4 reports all baseline comparisons. The published FCNN result (0.946) is a numerical reference only: without matched repeated runs or predictions, we cannot test significance against it. OpsSatNet-tiny reaches F1 =0.952±0.010 with approximately one sixteenth of the base model’s parameters.

Figure 3 complements the threshold-based comparison with ROC and precision–recall curves from representative fixed-epoch runs of OpsSatNet and all six raw-signal baselines. OpsSatNet and the convolutional alternatives retain high precision over most of the recall range, whereas the recurrent curves are less stable. This high-precision regime is operationally relevant because false alarms consume scarce operator attention.

### 4.6. Ablation Study

Table 5 compares full OpsSatNet with variants without FiLM, without attention (global average pooling), or without both. The full-minus-ablated mean F1 differences are 0.0064 for FiLM (95% CI: −0.0128 to 0.0256; p=0.408), 0.0056 for attention (−0.0161 to 0.0273; p=0.514), and 0.0096 for both (−0.0017 to 0.0209; p=0.077). All intervals include zero, so these five-seed results do not establish an independent benefit of either module or equivalence to the ablated variants. Training variability and architectural redundancy are possible explanations: convolutional features may already encode sensor type, and global average pooling may suffice for some segments. The effects need not be additive because the two modules can interact.

### 4.7. Computational Efficiency

Table 6 characterizes inference cost, and Figure 4 places every evaluated deep model on the F1–size plane. Desktop latency is measured on the hardware specified in Section 4.2. Each model uses FP32, evaluation mode, disabled gradients, batch size one, and input shape 1×1×256. Both PyTorch intra-operation and inter-operation thread counts are one. This is single-thread execution, not CPU-core affinity pinning. We use 100 warm-up passes followed by 1000 individually timed forward passes with a monotonic high-resolution timer; GPU timings synchronize CUDA after each pass. Timings exclude file loading, interpolation, normalization, host/device transfer, and plotting. Default-initialized models are used for timing because this dense architecture has a fixed execution graph.

Mean CPU latency is 1.157 ms for tiny and 3.251 ms for base; corresponding 95th percentiles are 1.204 and 3.340 ms. Standard deviations in Table 6 describe repeated calls in one session, not variability across hosts or operating conditions. FLOPs are estimated as twice the multiply–accumulate count from thop, including the attention scorer; this is a convention-based operation estimate rather than an exhaustive hardware instruction count. FP32 parameter storage is 65,192 bytes for tiny and 1,030,984 bytes for base, excluding buffers, activations, runtime, and workspace. Peak memory, energy use, worst-case execution time, and flight-hardware performance remain to be evaluated.

### 4.8. Per-Channel Analysis and Error Structure

Aggregate metrics can conceal channel-level failure modes, which matter operationally because each channel monitors a different subsystem. Figure 5 shows the test-set confusion matrix of a representative fixed-epoch OpsSatNet-base run and the per-channel F1 and error-rate breakdown. The model makes 12 errors among 529 segments: three false positives and nine false negatives. Most channels remain above F1 0.85, while PD6 is the most difficult channel in this run. Figure 6 shows the complete error set. Several missed anomalies resemble nominal smooth profiles at segment scale, whereas others contain localized bursts that the model scores below the fixed threshold. The errors therefore reveal both subtle-label ambiguity and genuine failure cases rather than supporting a claim of uniformly reliable channel-level detection.

For provenance, the qualitative figures use a separate archived fixed-epoch visualization run (seed 42, F1 =0.945), not the seed-42 run included in the five-run aggregate of Table 3 (F1 =0.973). Its 12 errors are therefore not the errors of an average or best run. This distinction applies to the OpsSatNet curves, error plots, t-SNE, and attention examples.

### 4.9. Cross-Type Generalization

Because the preceding results use joint training on both sensor types, we further test whether the learned representations generalize across types. The official training partition is first divided by the same deterministic channel–label stratification used for RNN tuning. Models are trained on one sensor type, checkpoints are selected only on that type’s training-derived validation subset, and the official in-type and unseen-type test subsets are evaluated once. We examine both transfer directions over five runs for OpsSatNet-base and the strongest raw-signal baseline, the plain CNN. For OpsSatNet, the unseen type’s FiLM embedding remains at its zero initialization, so the corresponding modulation is the identity transform. Table 7 reports the results.

Three observations emerge. First, a substantial domain gap separates the two types: every model loses F1 under zero-shot transfer, confirming the heterogeneity of the telemetry. The magnetometer-to-photodiode direction is especially asymmetric for OpsSatNet: zero-shot recall remains high (0.926), but precision falls to 0.561, indicating over-flagging of unfamiliar nominal photodiode profiles. Second, no model dominates both directions. Plain CNN is stronger for MAG → PD (F1 0.816±0.059 versus 0.682±0.125), whereas OpsSatNet is slightly stronger for PD → MAG (F1 0.867±0.029 versus 0.847±0.027). Thus, these results do not support a general claim that sensor conditioning improves zero-shot transfer. Third, joint training attains an overall F1 of 0.960 (Table 3), but this pooled score and the type-specific transfer scores concern different test populations and are not a matched estimate of the benefit of joint training. Deployment to a new telemetry type should therefore include representative labeled data rather than rely on type-agnostic transfer alone.

### 4.10. Qualitative Analysis

Figure 7 projects the attention-pooled feature vectors *z* of all test segments to two dimensions with t-SNE [43]. The displayed clusters are a qualitative visualization; the nonlinear projection does not establish linear separability in the original feature space.

Figure 8 shows one correctly classified anomalous segment from each channel with test anomalies. The profiles sometimes emphasize regions near spikes, discontinuities, or shape changes. These selected successes illustrate feature weighting but do not establish localization accuracy or explanatory faithfulness [39,40]. No interval-level anomaly annotations, perturbation-based faithfulness test, or operator study is used here.

## 5. Discussion

The results show that resampled raw telemetry contains sufficient morphological information for accurate segment-level classification on OPSSAT-AD. The plain CNN is already a strong baseline, consistent with the effectiveness of convolutional models for time-series classification [30,31]. OpsSatNet adds a multi-scale receptive field, type-dependent feature modulation, and attention aggregation, obtaining the highest mean F1 among the evaluated methods. The paired advantage over plain CNN is modest, and the individual effects of conditioning and attention are not statistically significant with five runs. The cross-type experiment further shows that conditioning does not guarantee superior zero-shot transfer in every direction.

Sensor type conditioning allows heterogeneous channels to share one feature extractor with a small mechanism for type-specific modulation. The cross-type experiment also shows the limit of that sharing: zero-shot transfer degrades substantially, especially from magnetometers to photodiodes. New sensor types should therefore contribute representative training data rather than rely on type-agnostic transfer alone. Attention profiles expose the learned aggregation weights; controlled perturbations, comparisons with other attribution methods, and operator evaluation are needed to assess their explanatory and practical value.

The tiny model combines 16K parameters, 8 MFLOPs, and F1 =0.952 in the reported setting. This makes it a candidate for resource-constrained sensor processing and motivates hardware evaluation. However, desktop single-thread latency does not establish onboard feasibility by itself. Flight deployment also depends on memory access, numerical precision, real-time scheduling, power consumption, radiation tolerance, and integration with existing telemetry software [2].

The dataset comprises telemetry from one CubeSat, with nine univariate channels and two sensor types; multivariate dependencies and cross-mission generalization remain untested. The curated 20.4% anomaly prevalence does not represent an operational event rate, and precision may fall at lower prevalence. Five seeds on one split provide limited evidence; the CNN comparison does not survive multiplicity correction, and the component ablations are inconclusive. The benchmark has been reused across model-development and protocol comparisons; final-epoch evaluation prevents per-run test-based checkpoint selection but cannot remove all benchmark-level selection effects. Recurrent models remain sensitive to training choices. Sample-index interpolation and instance normalization discard duration, irregular timing, offset, and scale that may contain diagnostic information. Binary segment labels do not identify precise intervals or physical fault causes. External evaluation on datasets such as ESA-ADB [11], rare-event prevalence studies, and flight-hardware measurements are required to assess broader applicability.

## 6. Conclusions

This paper presented OpsSatNet, a lightweight sensor-aware attention network for segment-level anomaly detection in satellite telemetry. On the official OPSSAT-AD split, the base model attains F1 =0.960±0.009, compared with 0.949±0.004 for the strongest retrained raw-signal baseline. The 16K parameter variant retains F1 =0.952±0.010 at 8 MFLOPs and 1.157 ms single-thread desktop CPU latency per segment. FiLM conditioning enables a shared backbone to adapt to magnetometer and photodiode signals, while attention pooling provides learned temporal aggregation weights. Overall, the results support compact, sensor-aware temporal modeling on OPSSAT-AD, while validation across missions, realistic anomaly prevalence, and flight-representative hardware remains necessary before operational deployment.

## Figures and Tables

**Figure 1:**
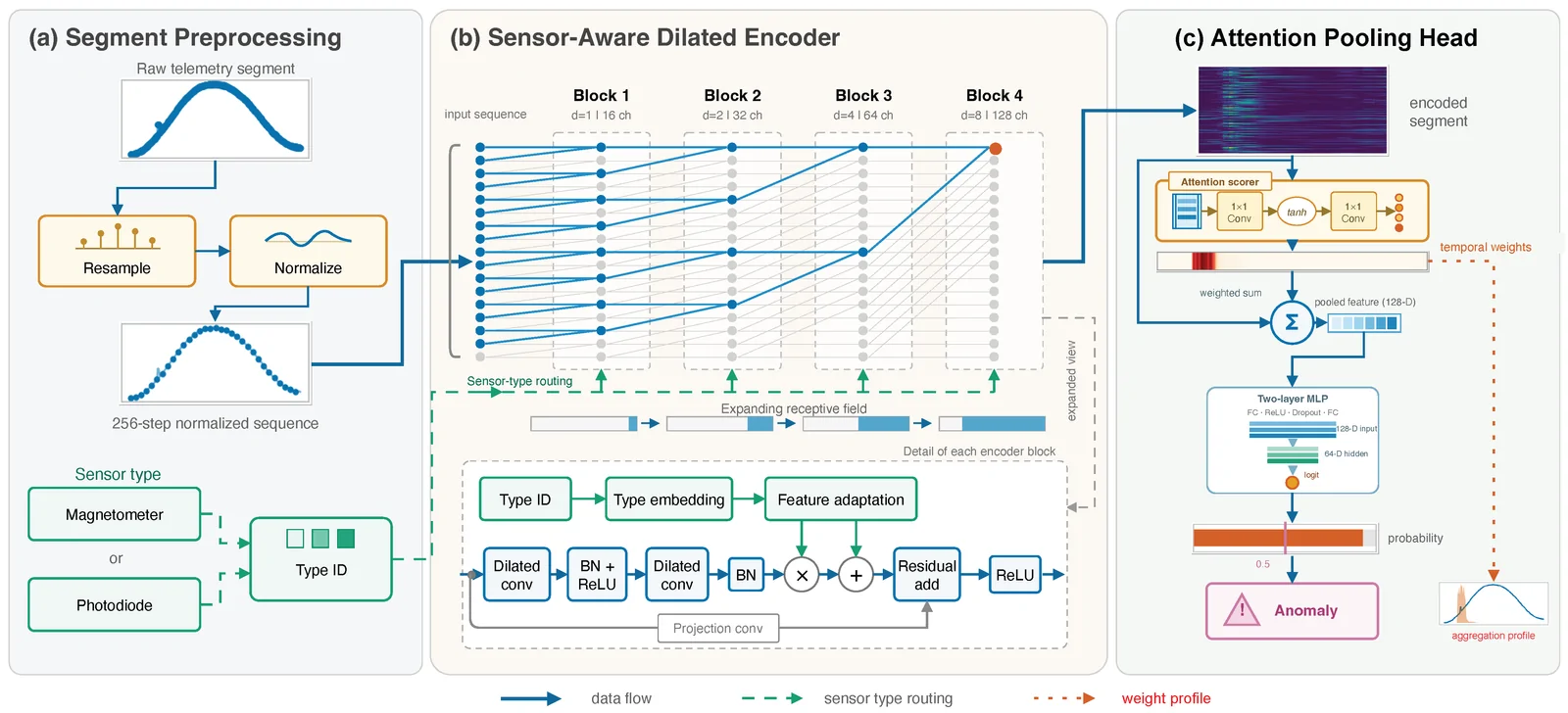
Overview of OpsSatNet. Variable-length telemetry is interpolated over sample index and normalized, with sensor type supplied as metadata. Dilated residual blocks extract temporal features, FiLM conditions these features, and attention pooling forms a weighted feature vector. The MLP produces an anomaly score.

**Figure 2:**
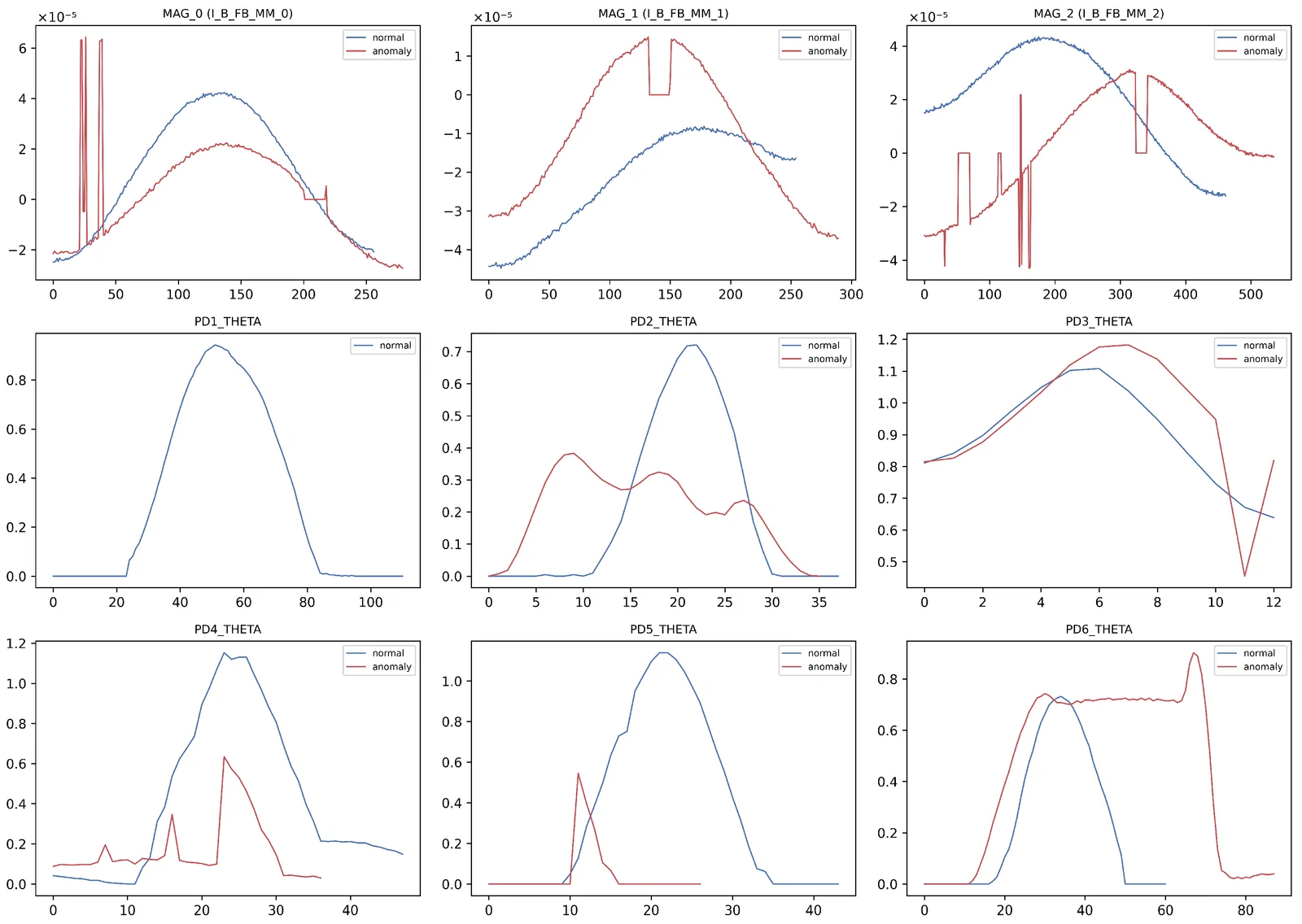
Representative nominal (blue) and anomalous (red) raw segments for each of the nine OPSSAT-AD channels. Horizontal axes show sample index; vertical axes show the telemetry values.

**Figure 3:**
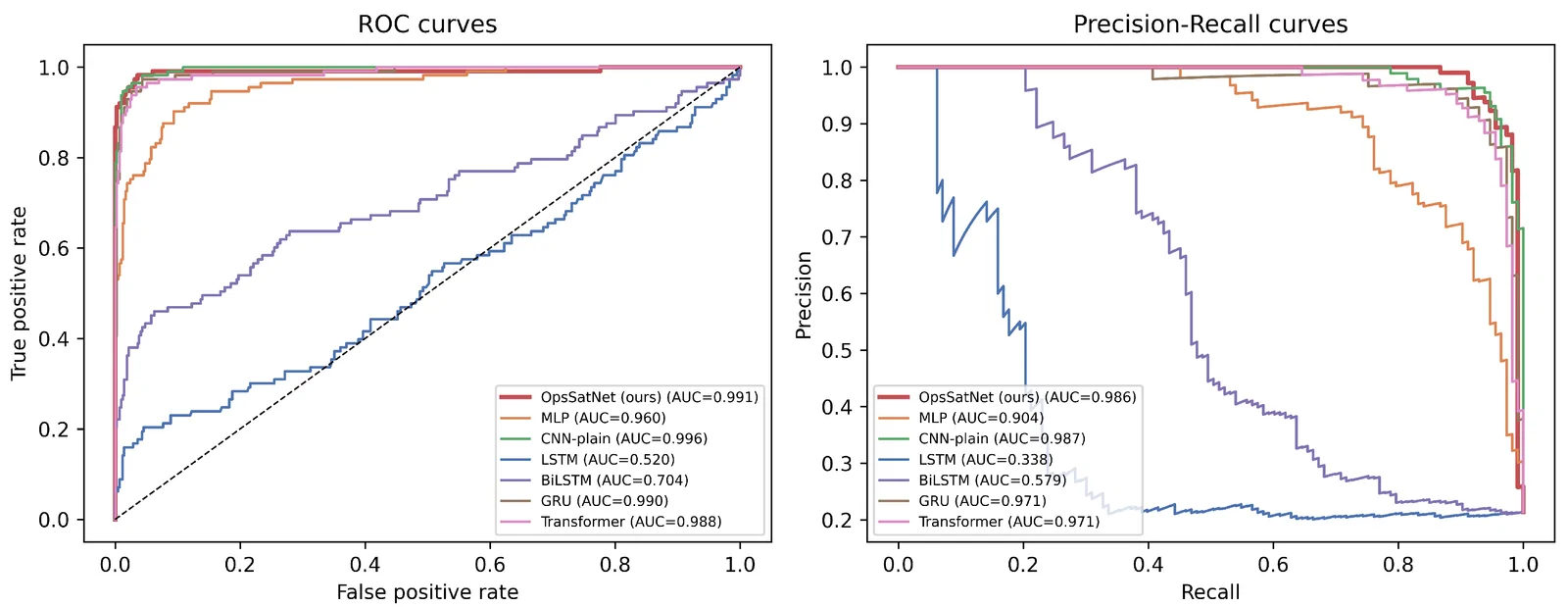
ROC curves (**left**) and precision–recall curves (**right**) on the official test split for one fixed-epoch run of OpsSatNet and the six raw-signal deep baselines. The curves use a uniform 60-epoch budget for visual comparability; the recurrent values in Table 3 instead use training-only validation selection. OpsSatNet remains competitive in the high-precision region.

**Figure 4:**
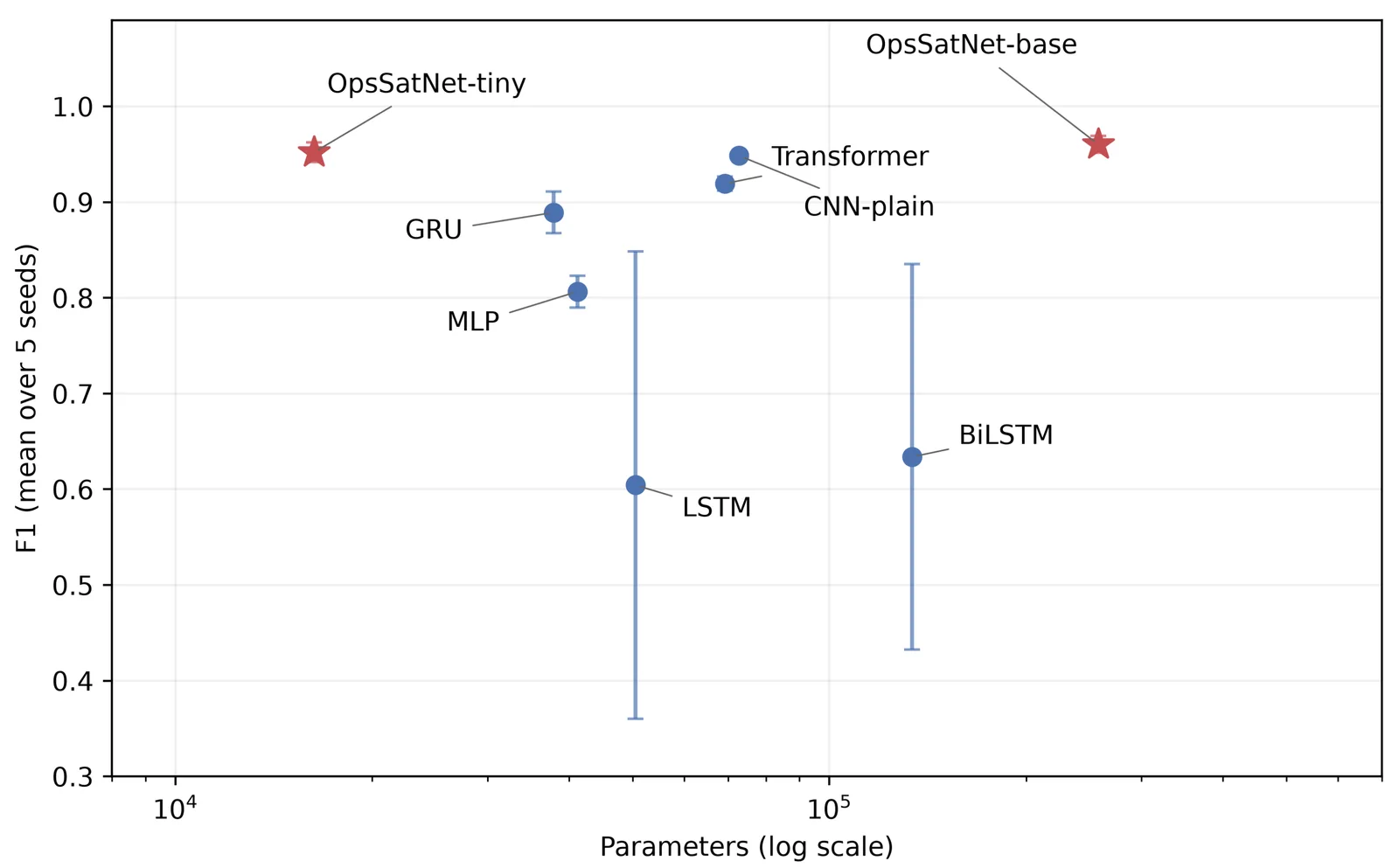
F1 versus model size for all raw-signal deep models (mean over 5 seeds; error bars show one standard deviation; logarithmic parameter axis). The two OpsSatNet variants (red stars) define the efficiency frontier.

**Figure 5:**
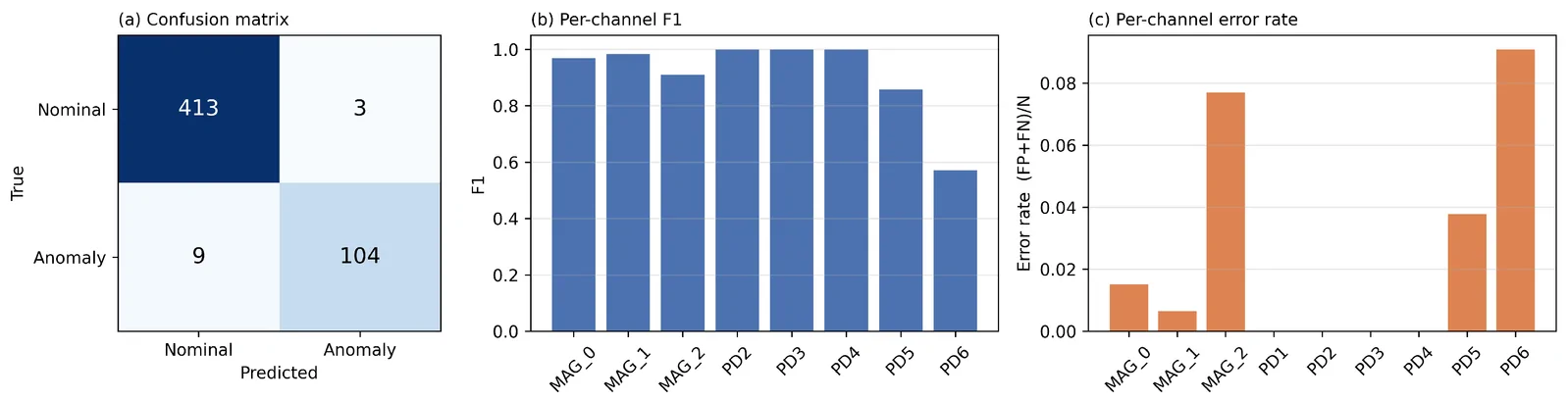
Error structure of OpsSatNet-base on the test split (seed 42, threshold 0.5). (**a**) Confusion matrix. (**b**) Per-channel F1 for the eight channels that contain test anomalies. (**c**) Per-channel error rate across all nine channels. Channels PD2 and PD4 contain very few test segments (fewer than a dozen each), so their per-channel statistics should be read as indicative only.

**Figure 6:**
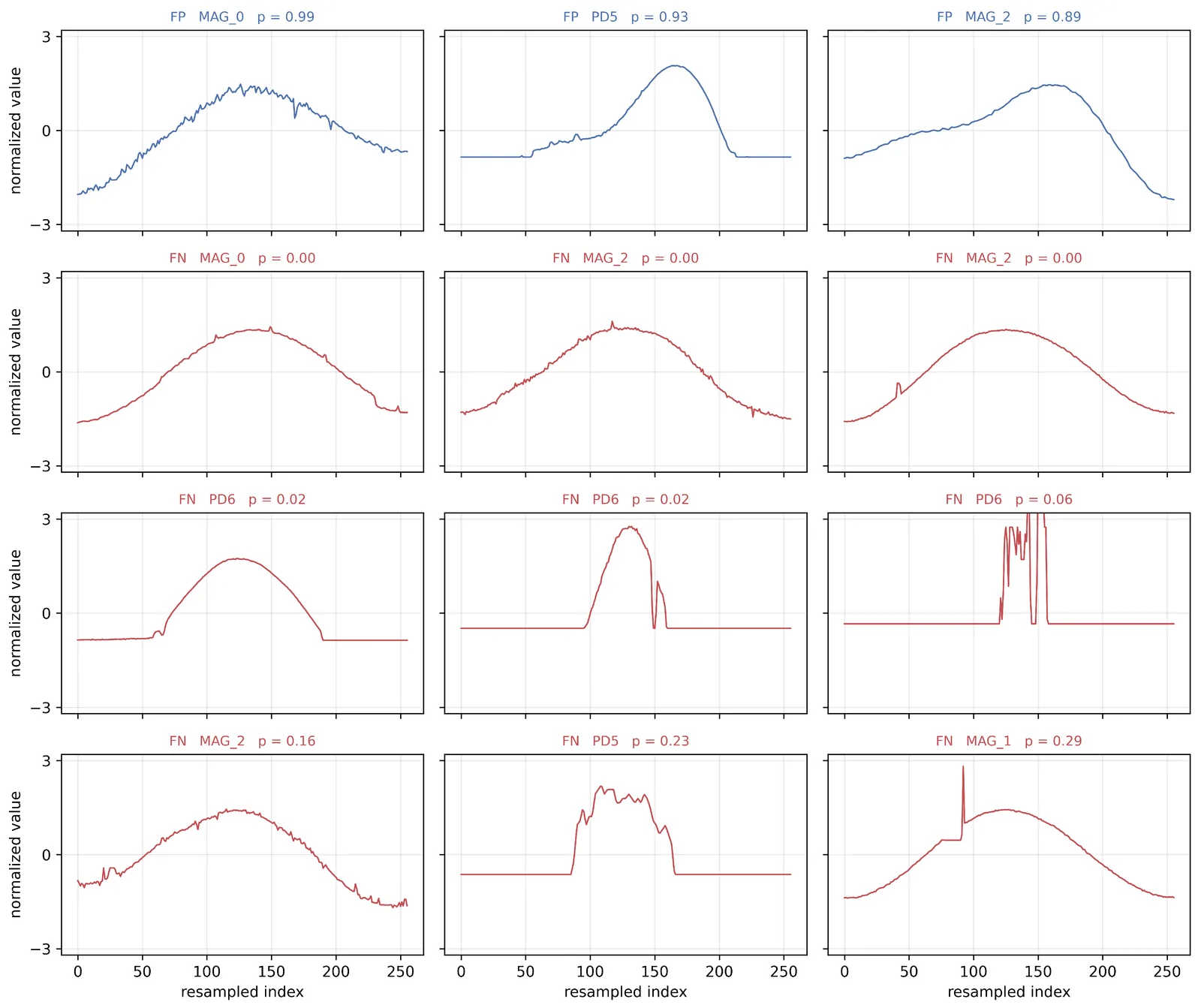
All 12 errors made by the representative fixed-epoch OpsSatNet-base run on the 529-segment test split: three false positives (blue) followed by nine false negatives (red), ordered by confidence within each group. Panels show the resampled, normalized signal consumed by the model, with a common vertical display range of [−3.2,3.2]; values outside this range are clipped. Predicted anomaly probability *p* is given per panel.

**Figure 7:**
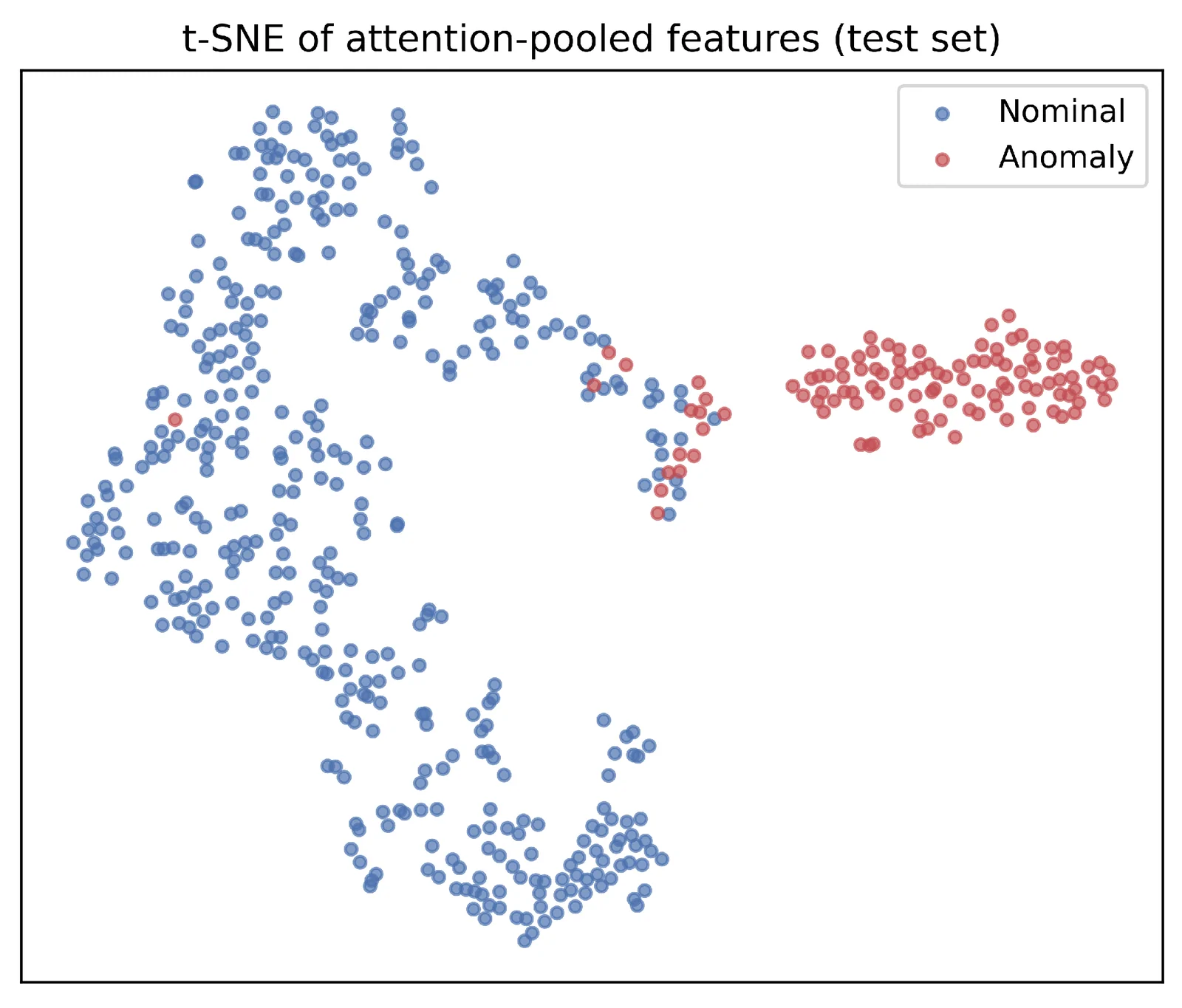
Two-dimensional t-SNE embedding of the pooled representation *z* produced by OpsSatNet-base for every test segment. This projection illustrates the separate visualization run described in Section 4.8; it does not establish linear separability in the original feature space.

**Figure 8:**
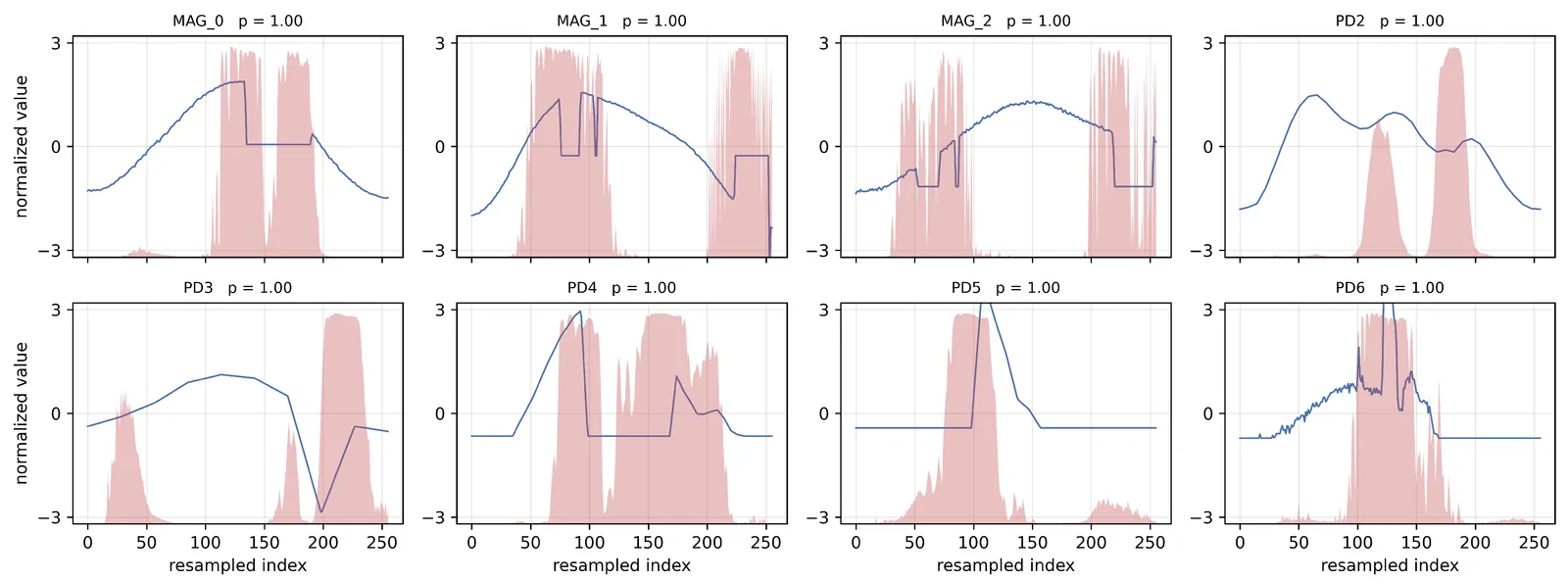
Descriptive attention profiles: weights α (red shading) overlaid on the normalized signal (blue) for one selected correctly detected anomaly per channel with test anomalies. Weights refer to encoded features with extended receptive fields, not validated pointwise anomaly labels. PD1_THETA is omitted because it has no test anomalies. Attention shading is scaled independently in each panel; signal traces share a vertical display range of [−3.2,3.2], with values outside this range clipped.

**Table 1:** Composition of the OPSSAT-AD dataset by telemetry channel (derived from the raw data; segment counts across both splits).

Channel	Sensor Type	Segments	Anomalous	Anomaly Rate
CADC0872 (I_B_FB_MM_0)	magnetometer	546	131	24.0%
CADC0873 (I_B_FB_MM_1)	magnetometer	593	105	17.7%
CADC0874 (I_B_FB_MM_2)	magnetometer	194	69	35.6%
CADC0884 (I_PD1_THETA)	photodiode	158	0	0.0%
CADC0886 (I_PD2_THETA)	photodiode	11	3	27.3%
CADC0888 (I_PD3_THETA)	photodiode	252	60	23.8%
CADC0890 (I_PD4_THETA)	photodiode	14	11	78.6%
CADC0892 (I_PD5_THETA)	photodiode	211	34	16.1%
CADC0894 (I_PD6_THETA)	photodiode	144	21	14.6%
Total	—	2123	434	20.4%

**Table 2:** OpsSatNet architecture settings and default training hyperparameters.

Hyperparameter	Value	Hyperparameter	Value
Resampled length *T*	256	Optimizer	AdamW
Batch size	64	Learning rate	$10^{-3}$ (cosine)
Epochs	60	Weight decay	$10^{-4}$
Loss	weighted BCE ($w_{+}\approx 4$)	Dropout	0.2
Kernel size *k*	7	Dilations	1, 2, 4, 8
Base widths	16–128 (*D* = 4)	Tiny widths	8–32 (*D* = 3)

**Table 3:** Results on the official OPSSAT-AD test split (529 segments). Published baselines are single-run results on 18 hand-crafted features from [1]; reproduced feature-based models use the reference seed; raw-signal models report mean ± standard deviation over five runs. OpsSatNet and non-recurrent raw-signal baselines use all official training data, a fixed 60-epoch budget, and one final test evaluation. ^1^ Recurrent baselines use gradient clipping and training-only validation for learning-rate and checkpoint selection, then evaluate the test set once. Best result per column is shown in **bold**.

Model	Input	Precision	Recall	F1	MCC	AUC-ROC
*Published feature-based baselines [1]*						
FCNN	18 features	0.963	0.929	0.946	—	0.989
XGBOD	18 features	0.944	0.894	0.918	—	0.992
RF + ICCS	18 features	0.978	0.805	0.883	—	0.985
MO-GAAL (best unsup.)	18 features	**0.985**	0.575	0.726	—	0.865
*Our feature-based reproductions*						
Random Forest	18 features	0.971	0.876	0.921	0.903	0.988
XGBoost	18 features	0.943	0.876	0.908	0.885	0.979
AdaBoost	18 features	0.918	0.788	0.848	0.814	0.963
MLP (feature-based)	18 features	0.946	0.619	0.749	0.721	0.959
*Raw-signal deep baselines (5 runs)*						
MLP (raw)	raw signal	0.900 ± 0.013	0.731 ± 0.024	0.807 ± 0.017	0.767 ± 0.018	0.951 ± 0.006
LSTM ^1^	raw signal	0.603 ± 0.306	0.628 ± 0.185	0.604 ± 0.244	0.482 ± 0.340	0.750 ± 0.163
BiLSTM ^1^	raw signal	0.635 ± 0.256	0.667 ± 0.188	0.634 ± 0.201	0.534 ± 0.268	0.778 ± 0.124
GRU ^1^	raw signal	0.858 ± 0.027	0.924 ± 0.021	0.889 ± 0.022	0.859 ± 0.028	0.976 ± 0.012
Transformer	raw signal	0.902 ± 0.024	0.938 ± 0.014	0.919 ± 0.007	0.897 ± 0.009	0.984 ± 0.006
CNN (plain)	raw signal	0.949 ± 0.009	0.949 ± 0.004	0.949 ± 0.004	0.934 ± 0.005	**0.995 ± 0.001**
*Proposed (fixed 60 epochs, 5 runs)*						
**OpsSatNet-base**	raw signal	0.978 ± 0.013	0.943 ± 0.015	**0.960 ± 0.009**	**0.950 ± 0.011**	0.989 ± 0.004
OpsSatNet-tiny	raw signal	0.951 ± 0.014	**0.954 ± 0.012**	0.952 ± 0.010	0.940 ± 0.013	0.991 ± 0.004

**Table 4:** Exploratory paired F1 comparisons between OpsSatNet-base and six raw-signal baselines (five seeds, one test split). Confidence intervals are unadjusted; Holm correction covers all six comparisons.

Baseline	Mean Difference (95% CI)	Raw *p*	Holm *p*
MLP	0.1538 (0.1371, 0.1705)	<0.0001	<0.0001
CNN	0.0118 (0.00004, 0.02356)	0.0495	0.0688
Transformer	0.0410 (0.0232, 0.0588)	0.0031	0.0153
LSTM	0.3560 (0.0628, 0.6492)	0.0280	0.0688
BiLSTM	0.3266 (0.0741, 0.5791)	0.0229	0.0688
GRU	0.0712 (0.0337, 0.1087)	0.0062	0.0249

**Table 5:** Ablation study (mean ± standard deviation over five seeds). SC = sensor conditioning; AP = attention pooling (vs. global average pooling).

Config	SC	AP	Accuracy	Precision	Recall	F1	MCC
base (full)	✓	✓	0.983 ± 0.004	0.978 ± 0.013	0.943 ± 0.015	0.960 ± 0.009	0.950 ± 0.011
– attention	✓	–	0.981 ± 0.006	0.956 ± 0.016	0.954 ± 0.013	0.955 ± 0.012	0.943 ± 0.015
– sensor cond.	–	✓	0.980 ± 0.005	0.954 ± 0.018	0.954 ± 0.015	0.954 ± 0.011	0.942 ± 0.014
– both	–	–	0.979 ± 0.002	0.961 ± 0.013	0.942 ± 0.016	0.951 ± 0.006	0.938 ± 0.007
tiny (full)	✓	✓	0.979 ± 0.005	0.951 ± 0.014	0.954 ± 0.012	0.952 ± 0.010	0.940 ± 0.013

**Table 6:** Desktop forward-pass cost (batch size 1, FP32). Latency is mean ± standard deviation across 1000 timed calls after 100 warm-ups.

Variant	Parameters	FLOPs	CPU Latency (ms)	GPU Latency (ms)
OpsSatNet-tiny	16,298	$7.97\times 10^{6}$	1.157±0.034	1.076±0.070
OpsSatNet-base	257,746	$1.27\times 10^{8}$	3.251±0.050	1.411±0.010

**Table 7:** Cross-type generalization (mean ± standard deviation over five runs). Models train on one sensor type; checkpoint selection uses only a training-derived in-type validation subset, and both official test subsets are evaluated once. Joint-training reference: OpsSatNet-base F1 =0.960 (Table 3).

Model	Direction	In-Type F1	Zero-Shot F1	Zero-Shot Precision	Zero-Shot Recall
OpsSatNet-base	MAG → PD	0.977 ± 0.012	0.682 ± 0.125	0.561 ± 0.164	0.926 ± 0.069
CNN (plain)	MAG → PD	0.932 ± 0.020	0.816 ± 0.059	0.754 ± 0.112	0.904 ± 0.042
OpsSatNet-base	PD → MAG	0.895 ± 0.021	0.867 ± 0.029	0.918 ± 0.036	0.823 ± 0.038
CNN (plain)	PD → MAG	0.904 ± 0.072	0.847 ± 0.027	0.828 ± 0.065	0.874 ± 0.062

## Data Availability

The OPSSAT-AD dataset analyzed in this study is publicly available from Zenodo at https://doi.org/10.5281/zenodo.12588359 under a CC-BY-4.0 license [21]. The implementation and scripts supporting the reported experiments are available from the corresponding author upon reasonable request and will be deposited in a public repository before publication.

## Abbreviations

The following abbreviations are used in this manuscript:

AUC	Area Under the Curve
BCE	Binary Cross-Entropy
CNN	Convolutional Neural Network
ESA	European Space Agency
FCNN	Fully Connected Neural Network
FiLM	Feature-wise Linear Modulation
FLOPs	Floating-Point Operations
GRU	Gated Recurrent Unit
LSTM	Long Short-Term Memory
MCC	Matthews Correlation Coefficient
MLP	Multilayer Perceptron
PD	Photodiode
PR	Precision–Recall
ROC	Receiver Operating Characteristic
t-SNE	t-Distributed Stochastic Neighbor Embedding
